# Influence of Turn Cycle Structure on Performance of Elite Alpine Skiers Assessed through an IMU in Different Slalom Course Settings

**DOI:** 10.3390/s22030902

**Published:** 2022-01-25

**Authors:** Carla Pérez-Chirinos Buxadé, Josep Maria Padullés Riu, Dani Gavaldà Castet, Michela Trabucchi, Bruno Fernández-Valdés, Sílvia Tuyà Viñas, Gerard Moras Feliu

**Affiliations:** 1 National Institute of Physical Education of Catalonia (INEFC), University of Barcelona (UB), 08038 Barcelona, Spain; cperezchirinos@gencat.cat (C.P.-C.B.); jmpadu@gmail.com (J.M.P.R.); dgavalda@maladetastudio.com (D.G.C.); bfernandez-valdes@tecnocampus.cat (B.F.-V.); stuya@gencat.cat (S.T.V.); 2 Val d’Aran School of Sports Technicians (ETEVA), 25598 Lleida, Spain; 3 Department of Condensed Matter Physics, University of Barcelona (UB), 08028 Barcelona, Spain; michela.trabucchi@ub.edu; 4 School of Health Sciences, TecnoCampus, Pompeu Fabra University, 08302 Barcelona, Spain

**Keywords:** inertial measurement unit, wearable sensor, sports biomechanics, kinematics, skiing, turn phases, accelerometer, magnetometer

## Abstract

Small differences in turn cycle structure, invisible to the naked eye, could be decisive in improving descent performance. The aim of this study was to assess the influence of turn cycle structure on the performance of elite alpine skiers using an inertial measurement unit (IMU) in different slalom (SL) course settings. Four SL courses were set: a flat-turned (FT), a steep-turned (ST), a flat-straighter (FS) and a steep-straighter (SS). Five elite alpine skiers (21.2 ± 3.3 years, 180.2 ± 5.6 cm, 72.8 ± 6.6 kg) completed several runs at maximum speed for each SL course. A total of 77 runs were obtained. Fast total times correlate with a longer initiation (INI) time in FT, a shorter steering time out of the turn (STE_OUT_) in the FT and FS and a shorter total steering time (STE_IN+OUT_) in the FT and SS courses. The linear mixed model used for the analysis revealed that in the FT-course for each second increase in the INI time, the total time is reduced by 0.45 s, and for every one-second increase in the STE_OUT_ and STE_IN+OUT_ times, the total time increases by 0.48 s and 0.31 s, respectively. Thus, to enhance descent performance, the skier should lengthen the INI time and shorten the STE_OUT_ and STE_IN+OUT_ time. Future studies could use an IMU to detect turn phases and analyze them using the other built-in sensors.

## 1. Introduction

In alpine skiing competitions, the skier must go through a course that forces the skier to make turns [1]. The ski turn is a fundamental technical element for speed regulation. In competition, the aim is to make turns while losing as little speed as possible, which makes it a subject of interest to both coaches and researchers.

It has been observed that differences in performance are more evident when analyzing the different phases of the turn, as these are more diffuse over the entire turn cycle. [2]. Thus, the division of the turn into its individual phases is essential to obtain a more detailed analysis of the performance of the turn. In the literature, the division of the turn cycle into its different phases is known as the turn cycle structure [3]. There is no consensus on the number of phases into which a turn should be divided. There are authors who have divided the turn into two [4,5,6,7], three [2,3,8,9,10] or four phases [11,12]. However, most studies agree on a fundamental division on two phases within the turn: the initiation (INI) phase, described as the motion between turns where lateral forces are non-existent, and the steering (STE) phase, characterized as the skier–ski system moving along a curvilinear trajectory with a centrifugal force acting on the system [13].

To determine the phases of a ski-turn on the basis of objective and comparable data, kinetic [4,5,7,8,14] and kinematic [2,6,9,11,15] criteria have been used.

Referring to kinetic criteria, the most widely used systems are pressure insoles, placed in the ski boot [7,8,16], and portable force plates, generally located between the bindings and the skis [5,14]. Both systems measure the interaction between the skier and the skis and use the measurement of the reaction forces against the ground to determine turn phases. Although force platforms have been considered as the reference system for kinetic measurements [17,18], they can affect the skier’s technique and comfort due to the added weight in the ski binding complex [18,19]. In contrast, pressure insoles are less invasive in this respect [16,17]. However, their positioning can be a handicap for those skiers that use boots with a thermo-molded liner, which adapts precisely to the foot for the best control of the ski, since placing these insoles could detract from this effect [16]. An additional limitation is that due to their placement, they can only measure the pressure between the plantar surface of the foot and the boot, rather than the actual force transmitted from the skier to the skis [18,20] and not always indicate the magnitude of ground reaction force accurately [18,19].

To determine turn phases using kinematic criteria, two of the most relevant systems according to the literature are video recording systems [2,11,15,21,22,23] and Global Navigation Satellite Systems (GNSS) [24,25,26,27]. These systems contributed significantly to the precise delimitation of the turn phases based on kinematic analysis of selected body segment positions. Video recording systems have been the reference system for kinematic measurements due to their accuracy [21]. In 2003, Supej and colleagues [28] delimited a ski turn by the turn switch point (TS point), defined as the point of intersection between the skier’s center of mass (CoM) line projected onto the snow surface and the average ski line. The TS point has been the reference in the literature to delimit consecutive turns and has helped later studies to define turn phases [2,3,11]. However, the complication of measuring with video recording systems in a natural environment, as well as the small number of turns that can be analyzed and the extensive time for post-processing the data, makes it difficult to collect data on a regular basis. Subsequent studies with wearable systems, such as GNSS receivers, have also proven to be valid for detecting the TS point [24,25,26] and turn phases [26]. Despite its validity, this system involves the skier wearing a small backpack with a receiver and an antenna placed on the helmet to allow better satellite visibility [27] on what could influence the skier’s technique [29]. In addition, the number of satellites and environmental obstructions can affect the quality of the recording data [27].

In recent years, IMUs have been revolutionizing training monitoring in alpine skiing [16,30,31,32]. They offer a number of advantages that go beyond the limitations of traditional or laboratory methods [30]: (a) work wirelessly; (b) small and lightweight; (c) easy to carry; (d) easy to place; (e) with the capacity to record a large number of data; (f) instantaneous data acquisition. In addition, the fact that all the built-in sensors (accelerometer, gyroscope, GNSS receiver, barometer, magnetometer, etc.) are synchronized optimizes data collection. In the field of alpine skiing, it has been demonstrated that the TS point can be detected with the use of a single IMU through the inclination of the pelvis [16], or data based on the lateral acceleration of the trunk [33] or the angular velocity of the boot [34]. The latter two have been two of the most accurate and precise methodologies [35].

Several studies have highlighted that in the turn cycle structure, the longer INI phase translates into better performance on the turn [2,36]. However, the influence on performance along a ski run (linked curves) has not been investigated yet. Furthermore, in such a changing environment as skiing, it is interesting to assess whether different course settings and steepness of the slope could influence the turn cycle structure or if contrary similar characteristics are observable regardless of the context. To our knowledge, no study has explored turn phases with an IMU or studied whether the turn phases can influence the performance of a whole run in different course settings. Therefore, the aim of this study was to assess the influence of turn cycle structure on total descent time of elite alpine skiers using an IMU in different slalom course settings.

## 2. Materials and Methods

### 2.1. Participants

Five elite alpine skiers (21.2 ± 3.3 years, 180.2 ± 5.6 cm, 72.8 ± 6.6 kg, 39.6 ± 7.6 slalom International Ski Federation (FIS) Points) participated in the study. Written informed consent was obtained from all participants. All the procedures were approved by the Ethics Committee for Clinical Sport Research of Catalonia (Study Number: 27/CEICGC/2020) and were conducted in accordance with the Declaration of Helsinki. 

### 2.2. Procedures

#### 2.2.1. IMU Device and Location

**Figure 1:**
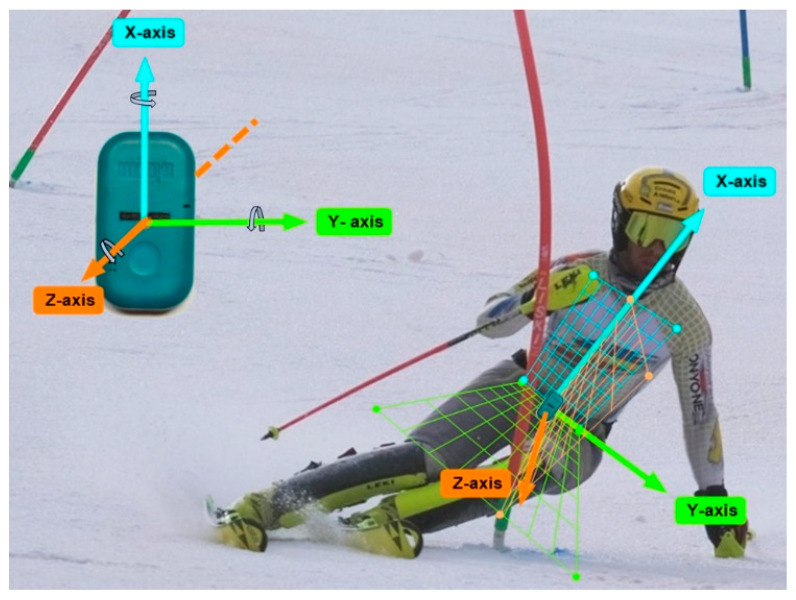
Measurement system and axis orientation. The *X*-axis (blue) is the vertical one, the *Y*-axis (green) is the lateral one, and the *Z*-axis (orange) is the antero-posterior one. Photo reproduced with permission from *Diana Martin i Gamisans/Crèdit Andorrà Supporting* and edited by the author.

An IMU device (WIMU, Realtrack Systems, Almeria, Spain; weight: 70 g; size: 81 mm × 45 mm × 15 mm) was used in this study. Signals from the 3-axial accelerometer (range: ± 400 G; sampling frequency: 1000 Hz) and the 3-axial magnetometer (range: ±8 gauss; sampling frequency: 100 Hz) were used. The IMU was also composed of a gyroscope, a barometer and a GNSS receiver, which were not used in this study. The IMU was attached to the lower back of skiers, at the L4–L5 level, using an adjustable sports lycra belt. This location close to the CoM has been shown to be the best place to assess whole body movement and to detect ski turns [16,37]. In addition, it is a comfortable place to wear for skiers, as they reported not noticing to wear it. The axes of the IMU device were defined as shown in Figure 1. Prior to the measurements, the calibration of IMUs was performed on a flat and even surface with the Z-axis perpendicular to the surface, according to the manufacturer’s specifications.

#### 2.2.2. Course Setting

**Figure 2:**
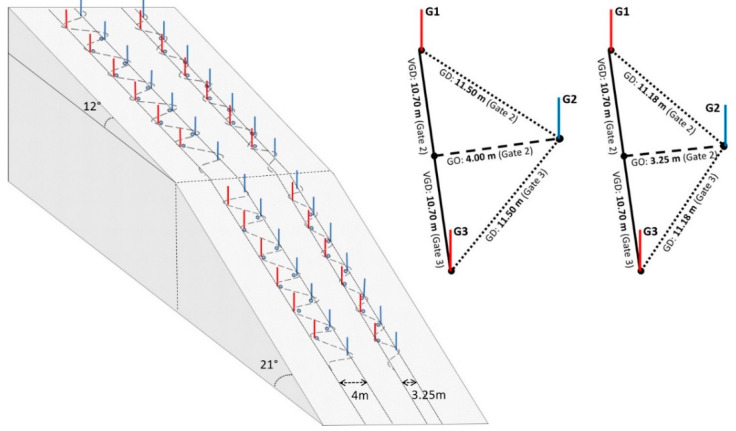
On-hill measurement setup. **Left**: schematic illustration of the four 10-gate SL courses. **Right** top: illustration of the course setting characteristics, characterized by gate distance (GD), gate offset (GO) and vertical gate distance (VGD).

Four different SL courses (Figure 2) were set according to the FIS rules [38]. The SL courses were chosen to cover 4 different scenarios in terms of slope (flatter: 12° and steeper: 21°) and type of trajectory (turned: 10.7/4 m course and straighter: 10.7/3.25 m course for vertical gate distance (VGD) and gate offset (GO), respectively) that can be experienced in certain sections during a slalom competition. For simplicity, a code has been given to each of the four SL courses according to their slope and trajectory: the turned course on the flatter slope (FT), the turned course on the steeper slope (ST), the straighter course on the flatter slope (FS) and the straighter course on the steeper slope (SS). The first letter of the code corresponds to the slope and the second letter to the trajectory. Ten consecutive gates were analyzed for each SL course. Tape measures were used to ensure that gates were placed equidistant from each other in each course. The courses located on the flatter slope (FT, FS) consisted of sixteen gates of which were analyzed from gates six (G6) to fifteen (G15), leaving gate sixteen (G16) at the end. The courses located on the steeper slope (ST, SS) consisted of thirteen gates of which were analyzed from gates nineteen (G19) to twenty-eight (G28), leaving gate twenty-nine (G29) at the end. Bar magnets (D33 mm × 267 mm, ND35, A.C. magnets 98, Barcelona, Spain) were placed on ten consecutive gates for each SL course, following the indications of Pérez-Chirinos Buxadé, et al. [39]. The first gate always turned to the right (left outer leg). 

Before starting, the skiers made a reconnaissance run down the courses. Skiers were then asked to complete three to five runs at maximum speed for each of the SL courses. A total of 77 runs were obtained: FT (n = 22), ST (n = 24), FS (n = 16) and SS (n = 15) from Skier 1 (n = 4 FT, 4 ST, 0 FS, 0 SS), Skier 2 (n = 4, 5, 4, 4), Skier 3 (n = 5, 5, 4, 4), Skier 4 (n = 4, 5, 4, 4) and Skier 5 (n = 5, 5, 4, 3). Skier 1 could not perform any descent of the FS and SS courses due to low back pain. A portable Full HD camera (Panasonic HC-V700) recording at 30 Hz was used to synchronize the accelerometry signal of the IMU to the videos of each run for a further visual checking. During data collection, the snow was hard-packed and groomed, and the coaches and experimental team smoothed the course prior to each and every run in an attempt to standardize conditions for side-skidding. Air temperatures progressively increased from 1.1 to 1.9 °C. Relative humidity decreased slightly from 71 to 61%. The maximum wind speed recorded was 6.3 km/h in a south-westerly direction, thus perpendicular to the direction of the ski runs.

#### 2.2.3. Drone Mission and Surveyed Reference Points

In order to generate a Digital Elevation Model (DEM) of the training area, a photogrammetric flight mission was planned using a drone (DJI Mavic Air, SZ DJI Technology Co., Hong Kong, China) equipped with the serial camera DJI, based on a 1.2/3” complementary metal-oxide-semiconductor (CMOS) sensor. After data collection with skiers, the flight mission was created with Drone Harmony Software (Drone Harmony AG, Zürich, Switzerland) that allowed for the creation of missions using an existing reference terrain model. The mission consisted of flying in grid format to complete 4 passes over the target area of 0.032 km^2^ and was planned with the following parameters: (a) flight speed 5 m/s (stopping and stabilizing before the shot); (b) ground sample distance 2.05 cm; (c) overlap and sidelap 70%; (d) camera angle 80°; (e) ground level of 55 m during the whole flight. The mission included two flights of 13 min 54 s and 8 min 06 s, respectively. A total of 76 high-resolution images were obtained. To provide reference points for the photogrammetry software, multiple precise (sub-centimeter accuracy) GNSS coordinates of all the gates in the four courses were surveyed using a Leica GS18 T RTK GNSS rover (Leica Geosystems AG, Heerbrugg, Switzerland). Additionally, the positions of 11 Ground Control Points were also surveyed (Supplementary Materials). 

### 2.3. Data Analysis

#### 2.3.1. Total Time

The skiers’ performance was evaluated with the total time of each run. The total time was defined as the time elapsed between the first and tenth gates and was calculated through a validated Magnet-Based Timing System (M-BTS) [39]. Magnets were placed on ten consecutive gates for each SL course, as explained above. The magnetometer built into the IMU detected the peak-shaped magnetic field when passing near the magnets at a certain speed. SPRO software (Realtrack Systems, Almeria, Spain) automatically calculated the time between the first and tenth peaks. 

#### 2.3.2. Criterion for Dividing Turns into Phases

For each run, the acceleration signal on the *Y*-axis coming from the SPRO software was filtered with a zero-lag, 4th order, low-pass Butterworth filter with a cutoff frequency of 6 Hz. This filter cutoff was chosen in order to maintain 95% of the signal power [40]. It was then exported to Microsoft Excel. A routine was programed in MatLab^®^ (The MathWorks, Natick, MA, USA) to separate INI and STE phases. The routine was programmed to detect the maximum acceleration peaks and the zero points of the acceleration signal closest to each side of the peak, which corresponded to the STE phase. The INI phase was then delimited between the STE phases of two consecutive turns.

**Figure 3:**
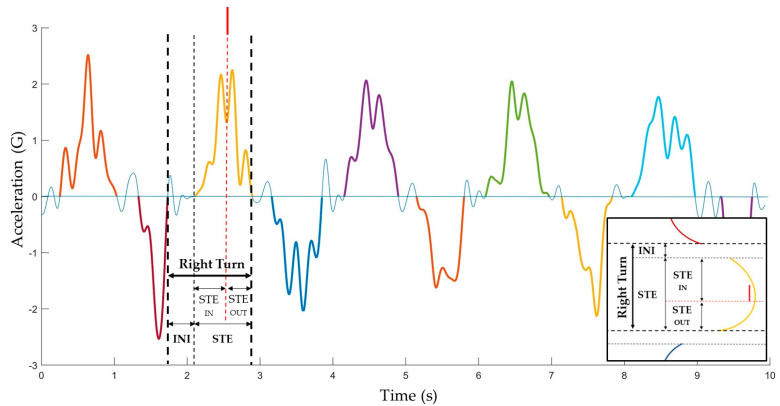
Graphical depiction of *Y*-axis acceleration and definition of turn phases: INI, initiation phase; STE, steering phase; STE_IN_, steering phase into the turn; STE_OUT_, steering phase out of the turn.

By plotting the *Y*-axis of the accelerometer values to the vertical axis of a graph, as shown in Figure 3, right and left turns could be identified. Positive peaks corresponded to right turns (left outer leg) and negative peaks to left turns (right outer leg). If the acceleration approaches the horizontal axis of the origin, as the horizontal axis represents a = 0 G, it means that acceleration on the lateral axis is decreasing. On the other hand, if it moves away from the horizontal axis, the acceleration is increasing. Unlike a previous study [33], we identified two main phases within the ski turn using an IMU device. INI phase was limited to the zone in which the acceleration on the *Y*-axis fluctuated around the zero-value corresponding to the motion between turns and all movements leading to the turn initiation. In this phase, the skier is practically with the CoM projected on the skis; therefore, there is no inclination of the skier’s trunk that produces an increase in the acceleration picked up by the *Y*-axis due to gravity. In contrast, the STE phase was limited to the zones where the acceleration on the *Y*-axis was moving away from zero, reaching a maximum peak and approaching zero again. This phase corresponded to the skier–ski system moving along a curvilinear trajectory with a centrifugal force acting on it and thus where lateral acceleration is present. 

By fusing the accelerometer information with an M-BTS that provides the position of the gate on the magnetometer signal [39], it was possible to divide the STE phase by the turn’s gate position [11] leading to the steering phases into the turn (STE_IN_) and out of the turn (STE_OUT_), where the acceleration on the *Y*-axis was moving away from zero or approaching zero, respectively (Figure 3).

Finally, for each run, the times corresponding to the same phase were added together, so the total time was decomposed into three partial times: INI time, time of STE_IN_ and time of STE_OUT_. The two steering times were added together to obtain STE time, hereinafter referred to as STE_IN+OU__T_ time. For each run, each of these partial times was expressed as a percentage of the total time.

#### 2.3.3. Digital Elevation Model (DEM)

In order to characterize the terrain and the different course settings, a DEM was created. A photogrammetry software (Agisoft Metashape Professional^®^ 1.5.2 version, Agisoft LLC, St. Petersburg, Russia) was used to convert footage from the drone to 3D virtual models. Initially, the program performs a photogrammetric triangulation from the images to identify common points between them and generate a dense cloud of points (46,283,238). This product was used to reconstruct the terrain and generate the initial DEM at a resolution of 4.06 cm/px, and an orthophoto map of 2.03 cm/px. These products were exported to ArcGIS free software (version 10.3, Environmental Systems Research Institute, Inc. Redlands, CA, USA), where a contour map (with 1 m equidistance) was generated from the initial DEM with a resolution of 4.06 cm/px. Afterwards, a slope map and an aspect map were also created at a resolution of 50 cm/px. All data can be accessed in the Supplementary Data file.

#### 2.3.4. Statistical Analyses

A total of 77 runs were analyzed. Despite having numerous runs from each skier for all courses, these are only repetitions from a sample of five. In this sense, the unit of analysis was the skier and the runs were repeated measures. Statistical analyses were performed to assess the influence of turn cycle structure (partial times) on the total descent time. To calculate the correlation, a Pearson’s linear correlation coefficient was used, and a scatter plot was made for each SL course. Each point represented the average of each skier’s runs. A Linear Mixed Model (LMM) was implemented for each partial time, where the skier corresponded to the random factor, the total time variable was the response variable and the partial time variable was the explanatory variable. The results reported correspond to the Analysis of Deviance Table (Type III Wald F tests with Kenward–Roger degrees of freedom [41]).

In addition, to explain the time differences more visually, the fastest and slowest runs for each skier on each SL course were selected. A bar graph was used to compare the mean of the fastest runs with the mean of the slowest runs for each SL run.

All database management tasks and statistical analyses were performed using R v4.0.4 software [42]. The significance level for all statistical tests was set at 5% (*p* < 0.05) or 10% (*p* < 0.1), indicated with an asterisk or with a dot, respectively.

## 3. Results

**Figure 4:**
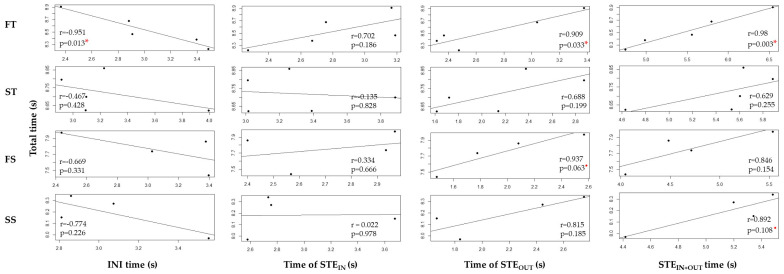
Correlations between the total descent time and the partial times (INI time, initiation time; time of STE_IN_, time of steering into the turn; time of STE_OUT_, time of steering out of the turn; STE_IN+OUT_ time, steering time) for four SL-courses (FT, flat turned course; ST, steep turned course; FS, flat straighter course; SS, steep straighter course). r, coefficient of correlation; *p*, level of significance at 5% or 10%, indicated with a red asterisk or a dot, respectively.

Figure 4 shows the results of the correlations between the total descent time and the partial times (INI time, time of STE_IN_, time of STE_OUT_ and STE_IN+OUT_ time). Each point represents the average of each skier’s runs.

By comparing INI time with total time, it was observed that as INI time increased, the total time decreased in all courses. The FT course had the highest and most significant correlation (r = −0.951, *p*-value = 0.013). In the FS and SS courses, the correlations between INI time and total time were high but not statistically significant (r = −0.669, *p*-value = 0.331 and r = −0.774, *p*-value = 0.226, respectively). Regarding the ST course, the correlation was weak and not significant (r = −0.467, *p*-value = 0.428).

The correlations between time of STE_IN_ and total time ranged from r = 0.702 (FT course) to r = 0.022 (SS course) and were not significant in any of the courses.

When comparing STE_OUT_ with total time, the longer the time of STE_OUT_, the longer the total time. The correlations between both times in the FT, FS and SS courses were 0.909, 0.937 and 0.815, respectively, with significance at 5% in the FT course and at 10% in the FS course. The weakest correlation was in the ST course (r = 0.688, *p*-value = 0.199).

Similarly, when comparing STE_IN+OUT_ time with total time, it was observed that by increasing STE_IN+OUT_ time, so did the total time in all courses. The correlations between both times in the FT, FS and SS courses were 0.98, 0.846 and 0.892, respectively, with significance at 5% in the FT course and at 10% in the SS course. The correlation in the ST course was moderate without statistical significance (r = 0.629, *p*-value = 0.255).

The linear mixed model used for the analysis revealed that INI time, time of STE_OUT_ and STE_IN+OUT_ time were good predictors of the total time in the FT course. Specifically, for every one-second increase in INI time, total time decreased by 0.45 s (LMM, F(1,3.40) = 6.61, *p*-value = 0.073), and for every one-second increase in STE_OUT_ and STE_IN+OUT_ times, the total time increased by 0.48 s (LMM, F(1,3.52) = 4.13, *p*-value = 0.025) and 0.31 s (LMM, F(1,3.34) = 11.76, *p*-value = 0.035), respectively.

**Figure 5:**
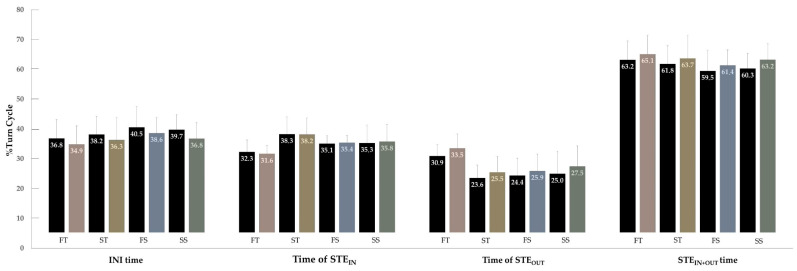
Comparison of turn cycle structure between Fast (black) vs. Slow (colored) runs for four SL courses: FT, flat turned course; ST, steep turned course; FS, flat straighter course; SS, steep straighter course. INI time, initiation time; Time of STE_IN_, time of steering into the turn; Time of STE_OUT_, time of steering out of the turn; STE_IN+OUT_ time, steering time. Values are means ± SD.

To visually highlight the differences between the fastest and slowest descents of each course, a bar graph was used (Figure 5). Although the results were purely descriptive, they complement the correlation analysis and the linear mixed model developed. The mean of the fastest and the slowest runs differed 2.5% on the FT course (8.43 ± 0.26 s vs. 8.64 ± 0.34 s), 3.3% on the ST course (8.56 ± 0.10 s vs. 8.84 ± 0.17 s), 2.4% on the FS course (7.68 ± 0.21 s vs. 7.86 ± 0.30 s) and 2% on the SS course (8.09 ± 0.17 s vs. 8.26 ± 0.19 s). Fast runs tended to have a longer INI time (FT: 1.95%, ST: 1.83%, FS: 1.90%, SS: 2.98%), a shorter time of STE_OUT_ (FT: −2.58%, ST: −1.93%, FS: −1.59% and SS: −2.49%) and a shorter STE_IN+OUT_ time (FT: −1.95%, ST: −1.83%, FS: −1.90% and SS: −2.98%), regardless of the course setting and steepness of the slope. In reference to the time of STE_IN_, similar results were observed between fast and slow descents. Another observation was that turned courses had a longer STE_IN+OUT_ time than straighter courses—concretely—time of STE_OUT_ predominated in the FT course and time of STE_IN_ predominated in the ST course.

## 4. Discussion and Conclusions

The purpose of this study was to assess the influence of turn cycle structure on the total descent time of elite alpine skiers using an IMU in different slalom course settings.

To our knowledge, this is the first study to date to determine turn phases by means of an IMU using kinematic criteria. A previous study already used lateral acceleration of the trunk to delimit linked turns in alpine skiing [33]. In this study, we wanted to follow this idea because of its high accuracy and precision [35]. However, for the present study, an accelerometer with a five times higher sampling frequency was used to clearly differentiate turn phases into the INI and STE_IN+OUT_ phases. Moreover, with the use of an IMU device, it is possible to detect the skier passing through the gates while performing a course [39]. This allowed for the incorporation of the position of the gate, with respect to the turn cycle structure, and to divide the STE_IN+OUT_ phase into STE_IN_ and STE_OUT_ phases.

The main finding was that regardless of course settings and steepness of the slope, lengthening the INI time and shortening the time of STE_OUT_ had an influence on improving descent performance. Time of STE_IN_ did not have any influence on total time.

With regard to INI time, the results obtained are in line with other findings. Spörri et al. [2] analyzed biomechanical aspects of giant slalom turns through a video-based system and observed that when comparing the fastest with the slowest turns in a 26/12 m course, the fastest had a 2.5% longer initiation in the turn cycle structure. In a similar way, Vaverka et al. [36], who analyzed left and right turns using a dynamometric system in a 14/4 m SL course, found that shorter duration turns had a 1.9% longer initiation. Unlike those mentioned above, the present study evaluated the influence of the turn cycle structure on the total descent time rather than on an isolated turn and was carried out in four different SL course settings. Fast runs tended to have a 1.95% longer INI time in the FT course, a 1.83% longer INI time in the ST course, a 1.90% longer INI time in the FS course and a 2.98% longer INI time in the SS course. The correlation between INI time and total time was high in the FT, FS and SS courses, with significance in the FT course (Figure 4). In addition, the predictive model was significant in the FT course, which indicated that for each second increase in the INI time, the total time is reduced by 0.45 s. This reduction of the total time by increasing the duration of the INI time is due to the fact that the lengthening of this phase optimizes the performance of the other phases by shortening them. Through the INI phase, the CoM increases speed until reaching maximal speeds close to the start of the STE_IN_ phase [3]. If we were to make the analogy with the motor world, the turn’s INI phase would be like stepping on the accelerator. For this reason, it may be of interest to lengthen this phase as much as possible but without negatively affecting the following turn phases.

Concerning the time of STE_IN_, it did not influence the total time. This fact could be explained because although the STE_IN_ phase is mostly towards the direction of the fall line [9], it is very likely that also includes a steering part out of the fall line since the STE phase was divided by the gate position and not by the fall line [11]. In the case of the FT course, there was a higher correlation than the other courses. A possible explanation could be that, in this case, most of the turn occurred before reaching the gate and that, therefore, the moment of the lowest skis’ turns radii and the highest energy dissipation occurred in the STE_IN_ phase [11,43], which was a flat terrain; this phase tended to decrease the total time. No article has been found that has discussed this phase and analyzed it from the point of view of the turn cycle structure. However, Spörri et al. [2] described a CoM direction change phase that would include the STE_IN_ phase and a part of the STE_OUT_ phase. They conclude that in a 26/10 m course, the CoM direction change phase was 2.7% shorter in fast turns and that there were no clear differences between fast and slow turns for the 26/12 m course.

Regarding the time of STE_OUT_, the results indicated that this was a decisive phase of the turns that influenced descent performance, especially in the flatter courses (FT and FS), where there was a very high significant correlation (Figure 4). In the FT course, the time of STE_OUT_ is a good predictor of total time; for every one second increase in STE_OUT_, total time increases by 0.48 s. As other authors have previously reported, this phase directs the skier–ski system away from the fall line [9] and, therefore, would be the equivalent of stepping on the brake. With the introduction of the carving technique, the time of STE_OUT_ was reduced compared to traditional parallel turns [8]. The STE_OUT_ phase defined in this study resembles the completion phase defined by Spörri et al. [2], where the fastest turn on a 26/12 m course had a 3.7% shorter completion. Similar results were reported in this study, where the fastest runs had a 2.58% shorter STE_OUT_ time in the FT course, a 1.93% shorter STE_OUT_ time in the ST course, a 1.59% shorter STE_OUT_ time in the FS course and a 2.49% shorter STE_OUT_ time in the SS course.

As for the STE_IN+OUT_ time, the results found were in accordance with those of Vaverka and colleagues, where the shortest duration turns had a shorter STE_IN+OUT_ phase [36]. Although they analyzed it for a turn, the same behavior was observed for a whole run. Fastest descents had a shorter STE_IN+OUT_ time in all of the courses (Figure 5). The highest and most significant correlations between total time and STE_IN+OUT_ time were observed in the FT and SS courses (Figure 4). In addition, in the FT course, STE_IN+OUT_ time was found to be a good predictor of total time. Specifically, a one-second increase in STE_IN+OUT_ time implies a 0.31 s increase in total time. The STE_IN+OUT_ phase defined in this study resembles the combination of the CoM direction change and completion phases defined by Spörri et al. [2]; however, a comparison with STE_IN+OUT_ is not feasible since Spörri analyzed both phases separately.

One of the features of the present study is the fact that it compares four different SL courses. Since the context of skiing is so changeable, it has been of interest to study how different slopes [22] and trajectories [2,3] have affected technical aspects. However, to the authors’ knowledge, this is the first study to consider steepness of the slope and trajectory together. In this study, in each SL course, the slope and trajectory were kept homogeneous in order to compare the four different scenarios. Terrain characterization is very important since, with it not being in a laboratory, it is necessary to control the external factors that can influence the results. For this reason, a DEM was generated to collect the variables of both the terrain and the configuration of the SL courses. It should be noted that capturing topographic data using drone photography to produce 3D models and DEMs has become more accessible and is an easier and more economical method for obtaining topographic data than traditional methods, such as real-time kinetic Global Positioning System mapping [44,45].

The conclusion of this study is that, regardless of the context, similar characteristics were observed in turn cycle structure for the fastest runs. In order to enhance descent performance, the skier should lengthen the INI time and shorten the STE_OUT_ time of the runs, thus reducing the STE_IN+OUT_ time. In general, straighter courses (FS and SS) had longer INI times and shorter STE_IN+OUT_ times than turned courses (FT, ST) and vice versa. It should be noted that for turned courses, the steepness of the slope had been the cause of the STE_IN_ time predominating, as in the case of the ST course, or the STE_OUT_ time predominating, as in the case of the FT course.

Small differences in turn cycle structure cannot be detected with the naked eye when a skier is descending at more than 40 km/h. This unnoticed aspect has proven to be decisive for performance when the differences between the best skiers are hundredths of a second. Therefore, the use of an IMU device will amplify the coach’s eye, allowing for the detection of small, but highly significant weaknesses.

## 5. Summary and Future Work

With the use of a single IMU device placed in the lower back of the skiers, it is possible to analyze the turn cycle structure that can help improve descent performance. Future applications could focus on the effect of other kinematic aspects coming from other sensors integrated in the IMU, including angular velocities, accelerations and even asymmetry detection that may have an influence on performance. It would also be interesting to analyze whether similar results in terms of turn cycle structure would also be found for other modalities, such as giant-slalom, super-G and downhill.

A limitation of the present study is that only five skiers were analyzed. This limits the generalizability of the study findings. However, in most of the literature that have used elite athletes in alpine skiing, studies with a single subject design are common, and very few exceed the sample size of this study. Therefore, the authors would like to emphasize the importance of having been able to obtain a sample of five elite alpine skiers to conduct the present study.

Our knowledge of the factors that determine performance in alpine skiing is currently relatively limited. The use of IMU devices, either individually or in combination with other systems, can help improve our understanding of this sport. Its user-friendly interaction is making it widely used not only in research settings, but also in the field to complement the analysis and feedback from coaches.

## Data Availability

Characteristics of terrain and course settings can be downloaded from Supplementary Materials.

## Supplementary Materials

The following are available online at https://www.mdpi.com/article/10.3390/s22030902/s1, Document S1: Characteristics of terrain and course settings.
