# Supplementary material for: Influence of Turn Cycle Structure on Performance of Elite Alpine Skiers Assessed through an IMU in Different Slalom Course Settings
Source: Sensors (Basel). 2022 Jan 25;22(3):902. doi: 10.3390/s22030902 (PMC8838443; doi:10.3390/s22030902)
Supplement: Supplementary file 1 [file sensors-22-00902-s001.zip › sensors-1557584-supplementary.pdf]

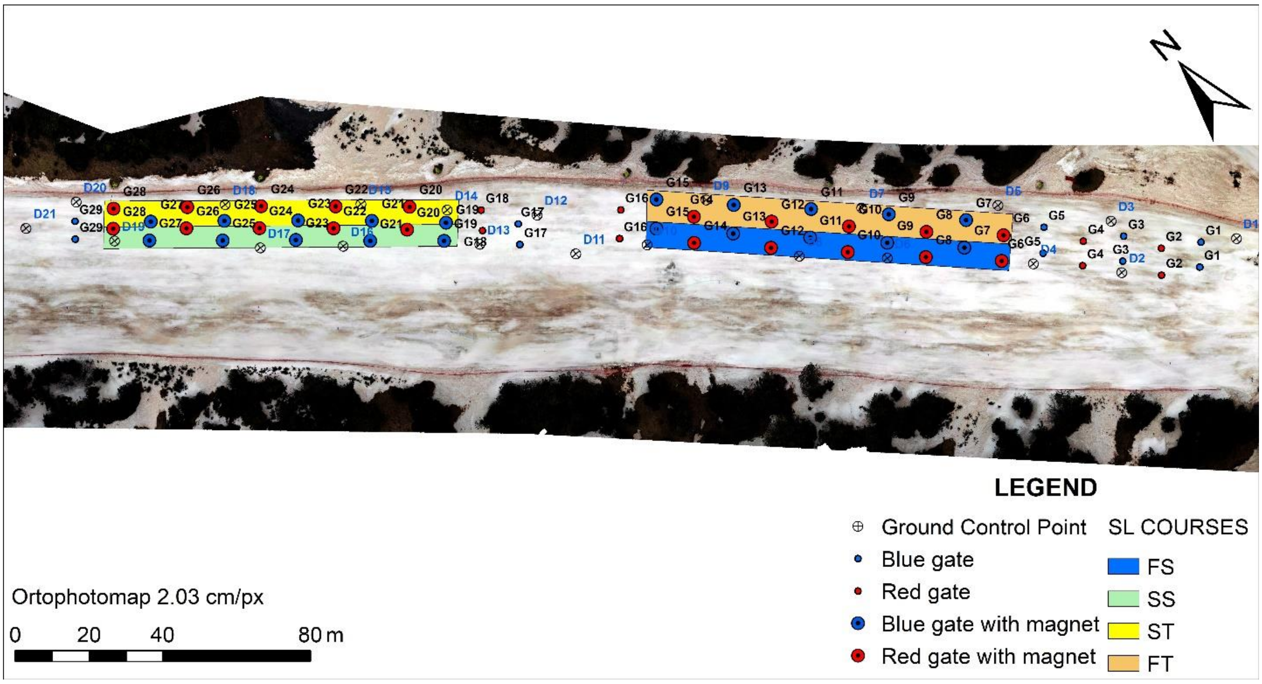

Figure S1. Orthophoto map generated by Agisoft® software. Four SL courses: FT, flat turned course (orange polygon); ST, steep turned course (yellow polygon); FS, flat straighter course (blue polygon); SS, steep straighter course (green polygon).

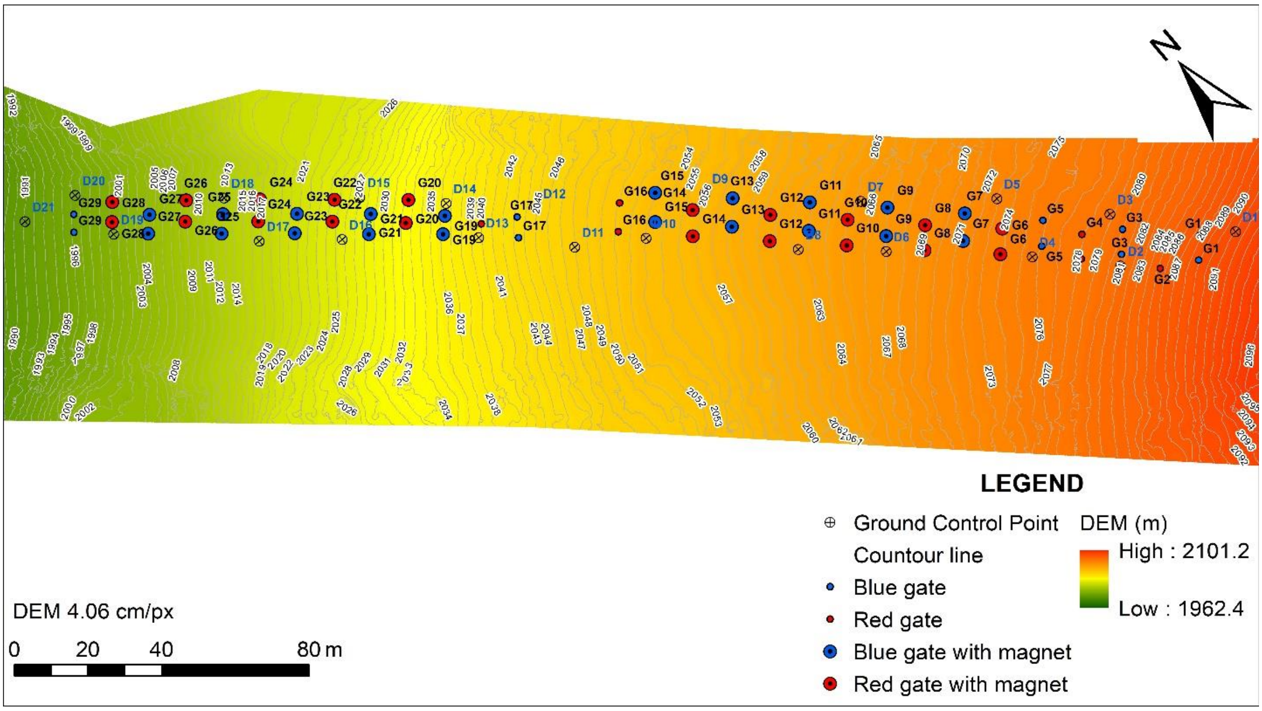

Figure S2. DEM with contour line map generated by Agisoft® software.

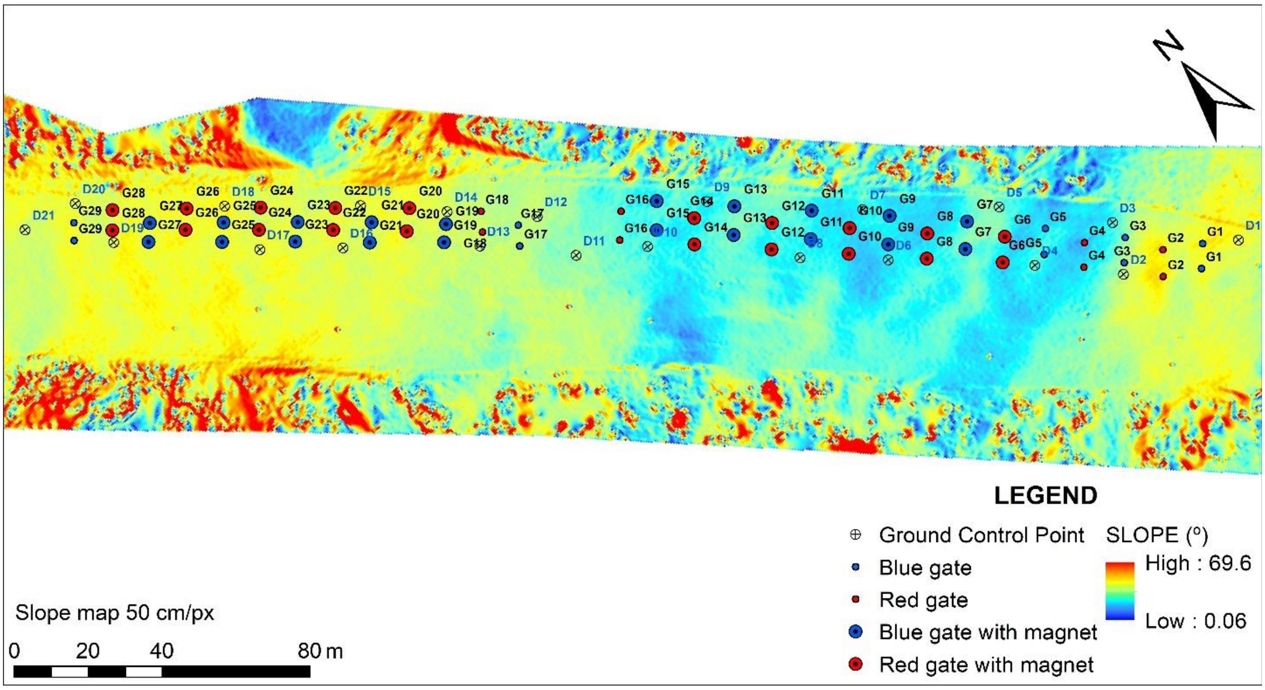

Figure S3. Slope map. For more information about how ArcGIS software calculates the slope see the following link: https://desktop.arcgis.com/en/arcmap/10.3/tools/spatial-analyst-toolbox/how-slope-works.htm

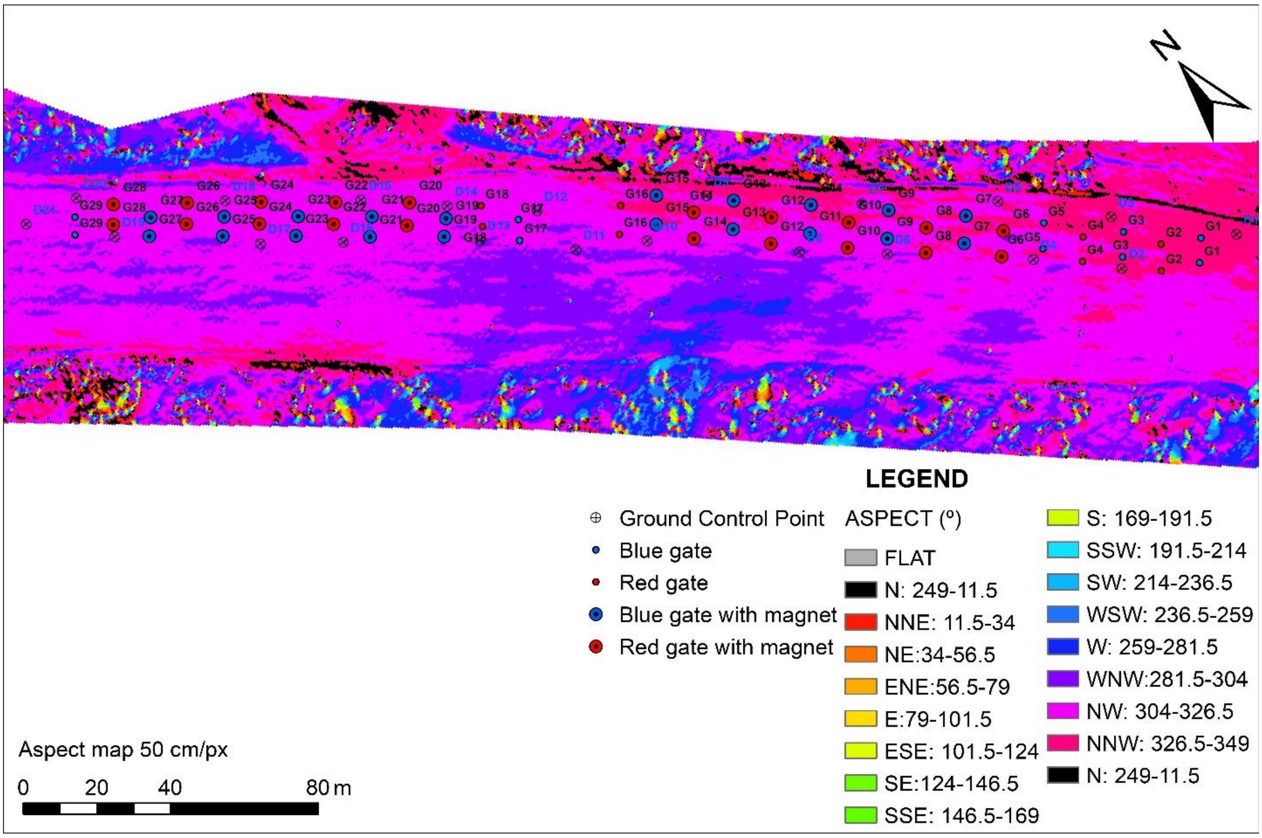

Figure S4. Aspect map. For more information about how ArcGIS software calculates the aspect see the following link: https://desktop.arcgis.com/en/arcmap/10.3/tools/spatial-analyst-toolbox/how-aspect-works.htm

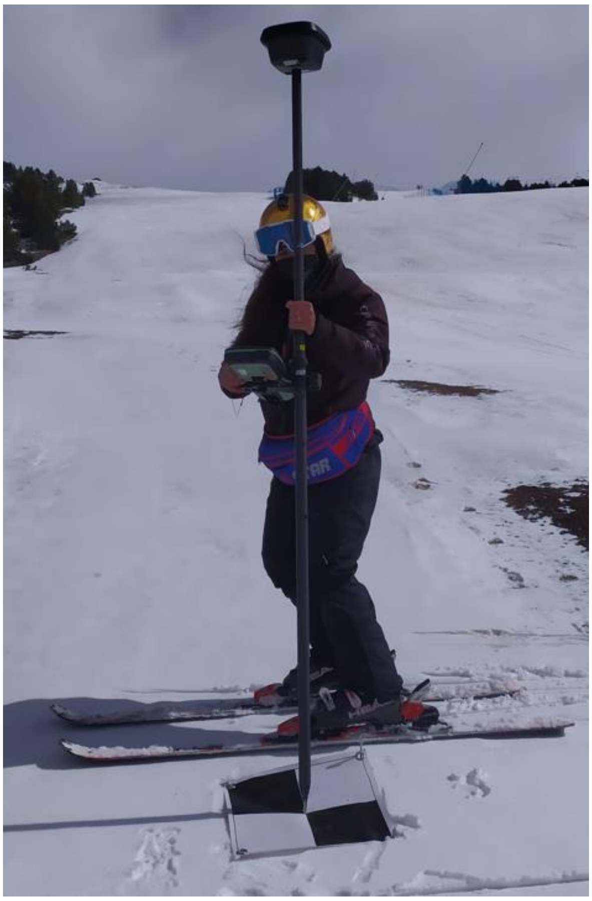

Figure S5. Ground control point position being captured using a Leica GS18 T RTK GNSS rover.

Table 1 – S1. Characteristics of the four SL course settings.

		Gate	Outer Leg	UTM Coordinates x (m)	y (m)	z (m)	Slope (°)	Aspect (°)	Cardinal Points	GD (m)	GO (m)	VGD (m)
**FT Course**	G6	1	L	389160.54	4713375.24	2073.86	13.22	320.23	NW	11.406	3.220	10.942
	G7	2	R	389155.40	4713384.93	2070.88	14.89	338.37	NNW	11.368	3.722	10.742
	G8	3	L	389145.06	4713389.39	2068.95	10.72	313.73	NW	11.421	4.012	10.694
	G9	4	R	389140.32	4713399.59	2066.88	11.99	326.07	NW	11.443	4.051	10.702
	G10	5	L	389129.90	4713404.03	2064.94	10.44	330.42	NNW	11.495	4.030	10.765
	G11	6	R	389125.09	4713414.24	2062.72	12.57	329.54	NNW	11.499	4.066	10.757
	G12	7	L	389114.70	4713418.54	2060.46	13.63	334.30	NNW	11.468	3.969	10.760
	G13	8	R	389109.76	4713428.58	2057.66	14.06	326.68	NNW	11.542	3.882	10.870
	G14	9	L	389099.40	4713433.10	2055.49	12.84	323.29	NW	11.505	3.941	10.809
	G15	10	R	389094.61	4713443.18	2052.94	13.39	329.52	NNW	11.452	3.674	10.846
**ST Course**	G19	1	L	389046.87	4713475.08	2036.40	20.94	307.78	NW	10.750	3.869	10.029
	G20	2	R	389042.05	4713484.75	2032.42	21.24	315.89	NW	11.515	4.064	10.774
	G21	3	L	389031.78	4713488.39	2028.51	22.27	317.24	NW	11.582	3.847	10.925
	G22	4	R	389026.73	4713497.69	2024.40	22.11	320.30	NW	11.347	3.850	10.674
	G23	5	L	389016.49	4713501.31	2020.66	21.21	313.05	NW	11.489	3.859	10.822
	G24	6	R	389011.30	4713510.76	2016.66	19.82	313.78	NW	11.503	3.956	10.801
	G25	7	L	389001.07	4713514.11	2012.74	23.21	313.70	NW	11.455	3.879	10.779
	G26	8	R	388995.80	4713523.42	2008.50	22.58	315.82	NW	11.500	3.825	10.845
	G27	9	L	388985.68	4713526.82	2004.49	21.79	311.64	NW	11.404	3.700	10.787
	G28	10	R	388980.18	4713536.00	2000.27	21.47	310.26	NW	11.505	3.464	10.971
**FS Course**	G6	1	L	389155.67	4713370.24	2073.85	9.59	322.31	NW	11.667	2.911	11.298
	G7	2	R	389150.25	4713379.50	2071.39	13.98	325.01	NW	11.012	3.104	10.565
	G8	3	L	389140.56	4713384.26	2069.20	11.95	317.82	NW	11.010	3.224	10.528
	G9	4	R	389135.07	4713393.93	2067.11	11.07	306.92	NW	11.317	3.206	10.854
	G10	5	L	389125.22	4713398.89	2065.19	10.18	319.98	NW	11.188	3.252	10.704
	G11	6	R	389119.92	4713408.50	2063.04	11.24	317.45	NW	11.185	3.379	10.662
	G12	7	L	389110.01	4713413.17	2060.75	12.49	305.36	NW	11.196	3.344	10.685
	G13	8	R	389104.65	4713422.75	2058.25	14.48	326.59	NNW	11.257	3.226	10.785
	G14	9	L	389094.87	4713427.62	2055.83	11.62	326.60	NNW	11.189	3.214	10.718
	G15	10	R	389089.53	4713437.14	2053.55	13.26	320.46	NW	11.147	3.242	10.665
**SS Course**	G19	1	L	389043.30	4713471.62	2036.25	20.00	306.02	NW	11.491	2.900	11.120
	G20	2	R	389037.52	4713480.42	2032.44	20.47	316.10	NW	11.194	3.037	10.775
	G21	3	L	389027.91	4713484.51	2028.49	20.11	308.03	NW	11.177	3.222	10.703
	G22	4	R	389022.48	4713493.50	2024.62	22.16	316.74	NW	11.191	3.282	10.699
	G23	5	L	389012.62	4713497.66	2020.66	22.50	307.67	NW	11.411	3.197	10.954
	G24	6	R	389007.12	4713506.48	2016.64	20.34	311.45	NW	11.146	3.279	10.652
	G25	7	L	388997.34	4713510.34	2012.70	21.94	306.88	NW	11.232	3.310	10.733
	G26	8	R	388991.90	4713519.20	2008.58	23.74	318.46	NW	11.179	3.287	10.685
	G27	9	L	388982.11	4713523.12	2004.51	21.65	302.21	WNW	11.299	3.223	10.829
	G28	10	R	388976.62	4713531.85	2000.38	21.13	314.27	NW	11.117	2.981	10.710

FT, flat turned course; ST, steep turned course; FS, flat straighter course; SS, steep straighter course; G, gate; L, left; R, right; UTM, Universal Transversal de Mercator; x, longitude; y, latitude; z, altitude; NW, Nord West; NNW, Nord Nord West; WNW, West Nord West; GD, gate distance; GO, gate offset; VGD, vertical gate distance.
